# Experiment with Light in the Treatment of Pulmonary Tuberculosis

**Published:** 1902-08

**Authors:** Robt. F. Williams

**Affiliations:** Richmond, Va.; Professor of Materia Medica and Therapeutics, Medical College of Virginia


### EXPERIMENT WITH LIGHT IN THE TREATMENT OF PULMONARY TUBERCULOSIS.*

By ROBT. F. WILLIAMS, M.A., M.D., RICHMOND, VA.

Professor of Materia Medica and Therapeutics, Medical College of Virginia.

My interest in light as a therapeutic agent was first stimulated eighteen months ago by the excellent results that I witnessed from the use of the arc light bath, with electricity, in a case of locomotor ataxia of several years' standing, and in a case of chronic articular rheumatism, where locomotion was almost lost.

In the beginning of my experiments with light my apparatus

---

* Read before the Richmond Academy of Medicine and Surgery, June 25, 1902.

consisted of an arc light bath. This was a cabinet lined with reflectors, in which the patient sat with the head out. In front and behind the patient were placed arc lamps of ordinary power, adjusted with a long arc so as to develop the violet ray. The temperature of this cabinet in a direct line between the lights was about 105° F. In other parts of the cabinet the temperature was about 97°. Here the patient (nude) was exposed to the rays from one-half an hour to one hour.

The immediate result of the bath was an increase of body temperature from one to two and a half degrees in spite of profuse sweating, which occurred usually in five to fifteen minutes. The pulse rate was increased and the tension softened. Though sweating was profuse, patients on lying down in a cooling-room invariably expressed themselves as feeling refreshed, though often tired when entering the bath. Other evidence of increased combustion than the rise of temperature was an increase of urea. In all of the early cases treated by this method I had a blood count made, and in all cases in which the count was less than normal, a second count was made after two weeks' treatment which showed in the different cases from twenty-five to forty per cent. increase in the red blood-cells. The white blood-cells were also increased though not by so great a percentage.

In connection with the arc light bath I used ozone, generated by fine electrical sparks from a high tension coil. This was arranged in a small cabinet in which the patient sat from five to fifteen minutes. I also used the high tension current directly on the body. Those cases were mostly neurasthenia and anemia, though I also treated two cases of locomotor ataxia. My results with the ataxia were negative, but in the nervous and anemic cases quite satisfactory. The case which I wish especially to report is that of a young man who gave the following history :

Age, twenty-two years; occupation for several years past, attendant at a soda-water fountain. He was sent to me in April, 1901, for treatment for gastric catarrh. He gave a history of mild neurasthenia for the previous several years. He also had several times trouble of the middle ear. He responded well to the treatment, and in a couple of months his stomach was in good condition and his nervous system showed improvement.

About the middle of September he began to feel bad and have a little continuing fever. On September 16 he consulted Dr. Frank M. Reade, who made a diagnosis of pulmonary tuberculosis and attended him until my return to the city on September 25 last. His fever was continuous, running as high as 103°. He had some night sweats, some cough, and lost a good deal of flesh. Physical examination showed only a thickening of the pleura at the base of the left lung. On September 29 we had the sputum examined and the bacilli were found. There was, however, no history of tuberculosis in the family. He was put on increasing doses of cod-liver oil, and sent to the country, where he stayed for a month.

On his return, the sweats had ceased and he had gained seventeen pounds; but the cough had increased, and, though morning temperature had disappeared, he was running an evening temperature of 100° to 101°.

Physical examination made November 18, on his return, showed the following: At the apex of the left lung and in the interscapular region dullness, fremitus equal to right lung, increased vocal resonance, whispered speech, broncho-vesicular breathing, prolonged expiratory sound, click at the end of forced inspiration, or, in other words, typical signs of consolidation of an area nearly the size of one's hand. The base of the left lung showed dullness to the level of the ninth rib in the scapular line extending to the front. Sounds on auscultation were diminished over this area. Deep breathing, commanded in the course of examination, invariably caused paroxysms of coughing which lasted several minute. These findings were verified by Dr. E. G. Williams and Dr. Frank M. Reade.

Treatment was then begun, consisting of arc light baths of thirty to forty minutes' duration ; ozone inhalations, which at first could be given only five or six minutes, as in that time it produced severe coughing, but which was increased until at the end of a month he could take it twenty minutes or longer without causing paroxysms. For the local stimulating effect I passed a current from the high tension coil directly through the diseased area for twenty minutes.

This treatment was daily. All medication was stopped, his occupation was changed, and he became a collector, under orders to walk at least five miles a day in all kinds of weather. He was also directed to sleep with his windows wide open.

After two days' treatment his temperature became normal and remained so continually. At the end of a month physical examination was again made by Dr. Reade and myself. We thought there was some diminution in the dullness and fremitus over the affected area, but of this we could not be sure. The click on forced respiration had disappeared and coughing was not excited at all by this effort. His appetite was excellent.

Dr. H. H. Levy examined him for me at this time and found dullness, prolonged expiratory note, subcrepitant râles, broncho-vesicular breathing at the apex and upper interscapular region and friction sounds at the base. I did not have a blood count made at the beginning of his treatment, though from clinical evidence he was unmistakably very anemic. On December 23d a blood count was made on coming to my office by Dr. E. G. Williams which showed 5,160,000 red corpuscles, 8,700 white corpuscles and hemoglobin ninety-three to ninety-five per cent. The count was again made immediately after he left the cabinet, when the red blood-corpuscles numbered 6,310,000, while the white blood-corpuscles showed 9,250. At this time he was leading an active life and expressed himself as feeling better than he had in several years.

Physical examinations were made again on January 13th and February 12th, at which the signs previously noted were found, though evidently less marked.

On February 15th this course of treatment was stopped, and treatment by Finsen light inaugurated.

My apparatus, called an actinolyte, manufactured by a New York firm, consists essentially of an enormous arc light, in which the carbons are set at right angles so as to save loss, the light from which is concentrated by a series of large lenses, giving, when reduced to a small focus, a light approximated at seventy to eighty thousand candle-power. The heat from this, which is so great that a cigarette can be lighted in the focus, is cut out by a water-bath, in which I have used methylen blue with best results.

The patient was stripped to the waist and placed about eight feet from the machine, with his back to the light, and the light focused to a circle that would cover the consolidated area, including the apex and upper half of the scapula. The sittings were thirty minutes daily. Ozone and electricity were stopped. Later the patient was reversed, the light being directed on the front of the affected area.

That this light penetrated the lung I demonstrated by an experiment previously frequently made. A negative was placed over the pectoral muscles and over that a sensitive plate, all of which was then covered with material impervious to light. The light was then turned on over the scapula for twenty minutes when the plate, on development, showed clearly a positive, which could only have been produced by rays passing through the negative after penetrating the body.

On March 19th physical examination showed a practically normal lung, with an occasional friction sound near the apex. In this finding Dr. Levy agreed.

On March 23d bacilli were still present, but the patient coughed only in the morning on arising, while taking deep-breathing exercises.

Treatment was continued with gradual lessening of the cough until the last week in April, when he told me that he had ceased to cough entirely.

About April 25th the sputum was examined and no bacilli were found. This demonstrated clearly the greater efficiency of the concentrated light in deep-seated organs as compared with the diffused light of the arc light bath.

About May 17th, after great exposure to the sun during the hot spell then on us, the patient complained of malaise, aching in the back, dull headache, etc. Thinking that he had taken cold I gave him some simple remedy which relieved the symptoms, except the headache.

On Tuesday, the 20th, he sent for me and I found him suffering from nausea and intense headache through the temples and cheek bones. His temperature was 100° F. He vomited once that day and once the next day. After that continued intense pain through the temples and face which resisted all forms of treatment, but anodyne and counterirritant; was the only symptom except earache. On examining the ear, without a speculum, I saw what seemed to be a large piece of wax which was removed by hydrogen dioxide, but the earache continued two or three days longer.

Dr. H. H. Levy saw him with me about May 26th, and, while of course suspecting meningitis, we concluded that it was a case of cerebral congestion. His temperature by this time was normal. He continued, with no other symptoms, in this condition until June 3d. Treatment consisted of cathartics, potassium iodide, atropine, ergot, ice-bag to the head, blisters to the back of the head and neck and leeches to the temples and behind the ears. He then developed symptoms of meningitis, spasm of groups of muscles, sluggish iris reflexes, stiffness and tenderness at the back of the neck, intense agonizing pain through the temples, difficulty or inability to speak. During this time Dr. Reade attended him with me. This condition lasted until June 6th, when he died.

Whether the meningitis was of tubercular origin or a simple meningitis from his nasal catarrh, ear trouble or exposure to the sun, can never be known, as an autopsy was not procurable.

In view of the patient's death nothing can be claimed as demonstrated, for, if he had lived, I should have considered an interval of many months without return of his pulmonary symptoms as necessary to a demonstration of cure, but from the observations recorded in this case and the reports of other observers, I think the conclusion can at least be reached that it offers great hope in this affliction, perhaps more than any other single line of treatment, and that if perhaps associated with wise medication, as well as hygiene, its development may soon rob tuberculosis of its terror as antitoxin has done for diphtheria.

*S. E. Grace St.*

